# Honey: A Novel Antioxidant

**DOI:** 10.3390/molecules17044400

**Published:** 2012-04-12

**Authors:** Omotayo O. Erejuwa, Siti A. Sulaiman, Mohd S. Ab Wahab

**Affiliations:** Department of Pharmacology, School of Medical Sciences, Universiti Sains Malaysia, 16150 Kubang Kerian, Kelantan, Malaysia; Email: sbsamrah@kb.usm.my (S.A.S.); msuhaimi@kb.usm.my (M.S.A.W.)

**Keywords:** honey, antioxidant, oxidative stress, chronic diseases, kidney, pancreas, liver, plasma, testis

## Abstract

The global prevalence of chronic diseases such as diabetes mellitus, hypertension, atherosclerosis, cancer and Alzheimer's disease is on the rise. These diseases, which constitute the major causes of death globally, are associated with oxidative stress. Oxidative stress is defined as an “imbalance between oxidants and antioxidants in favor of the oxidants, potentially leading to damage”. Individuals with chronic diseases are more susceptible to oxidative stress and damage because they have elevated levels of oxidants and/or reduced antioxidants. This, therefore, necessitates supplementation with antioxidants so as to delay, prevent or remove oxidative damage. Honey is a natural substance with many medicinal effects such as antibacterial, hepatoprotective, hypoglycemic, reproductive, antihypertensive and antioxidant effects. This review presents findings that indicate honey may ameliorate oxidative stress in the gastrointestinal tract (GIT), liver, pancreas, kidney, reproductive organs and plasma/serum. Besides, the review highlights data that demonstrate the synergistic antioxidant effect of honey and antidiabetic drugs in the pancreas, kidney and serum of diabetic rats. These data suggest that honey, administered alone or in combination with conventional therapy, might be a novel antioxidant in the management of chronic diseases commonly associated with oxidative stress. In view of the fact that the majority of these data emanate from animal studies, there is an urgent need to investigate this antioxidant effect of honey in human subjects with chronic or degenerative diseases.

## 1. Introduction

In the last couple of decades, there has been an increase in the global prevalence of degenerative or chronic diseases such as diabetes mellitus, hypertension, cancer, Alzheimer's disease, atherosclerosis and heart disease [1,2]. These diseases are now the major causes of death globally [1,2]. Recent evidence implicates the role of oxidative stress in the pathogenesis and/or complications of these disorders [3,4]. Oxidative stress is defined as an “imbalance between oxidants and antioxidants in favor of the oxidants, potentially leading to damage” [5]. It is caused by increased production and/or reduced removal of reactive species by the antioxidant defenses. Oxidative stress causes oxidative damage - “the biomolecular damage caused by attack of reactive species upon the constituents of living organisms” [6]. Oxidative damage to cellular components impairs physiological functions. Reactive species can be reactive oxygen species (ROS) or reactive nitrogen species (RNS) [5,6]. Reactive oxygen species include superoxide (O_2_^•−^), hydroxyl (^•^OH) and hydrogen peroxide (H_2_O_2_), while RNS are nitric oxide (NO), nitrogen dioxide (NO_2_^•−^) and peroxynitrite (OONO^−^) [5,6]. They are produced by aerobic organisms as byproducts of metabolism such as during mitochondrial electron transport chain or as a result of accidents of chemistry such as the autoxidation of unstable biomolecules (dopamine) [5,7]. Reactive species may also be produced in response to inflammation during which phagocytes release ROS to kill invading bacteria [5,7]. The ability of cells to scavenge excess reactive species is largely dependent on the efficiency of the overall antioxidant defense system [6,7]. This antioxidant defense network consists of endogenous and exogenous antioxidants. The endogenous antioxidants comprise the enzymatic antioxidants such as superoxide dismutase (SOD), catalase (CAT) and glutathione peroxidase (GPx) and non-enzymatic antioxidants including glutathione (GSH), vitamins C and E as well as small molecules [6]. The exogenous antioxidants comprise the micronutrients and other exogenously administered antioxidants [5,6]. As defined by Halliwell and Gutteridge, an antioxidant is “any substance that delays, prevents or removes oxidative damage to a target molecule” [6]. Available evidence indicates that individuals with chronic or degenerative diseases are more susceptible to oxidative stress and damage because they have elevated levels of oxidants and/or reduced antioxidants [3,4]. Therefore, it has been posited that antioxidant supplementation in such individuals may be beneficial [8,9,10].

**Figure 1:**
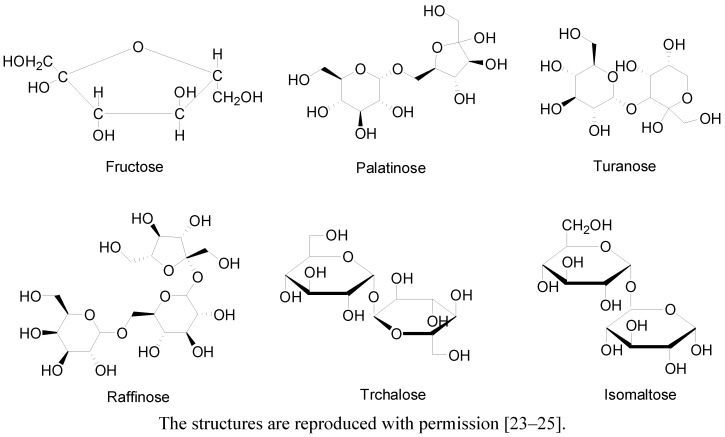
Molecular structures of fructose and oligosaccharides present in honey.

Honey, a natural product formed from nectar by honeybees, has been a subject of renewed research interest in the last few years. Evidence indicates that honey can exert several health-beneficial effects such as gastroprotective [11], hepatoprotective [12], reproductive [13,14], hypoglycemic [15], antioxidant [15], antihypertensive [16], antibacterial [17], anti-fungal [18] and anti-inflammatory [19] effects. It consists of primarily sugars such as monosaccharides, disaccharides, oligosaccharides and polysaccharides [20,21,22]. It contains enzymes such as glucose oxidase, diastase, invertase, catalase and peroxidase [20]. Honey also contains other bioactive constituents such as organic acids, ascorbic acid, trace elements, vitamins, amino acids, proteins and Maillard reaction products [20]. This review presents a synopsis of findings that indicate honey is a novel antioxidant. The data presented suggest that honey, administered alone or in combination with conventional therapy, might be of therapeutic benefits in the management of chronic diseases commonly associated with oxidative stress. This paper, which is a comprehensive review of the current literature, highlights the (potential) beneficial effects of honey based on its ability to ameliorate oxidative stress in different tissues, organs, body fluids or compartments. These include gastroprotective, hepatoprotective, renoprotective as well as its protective effect in the pancreas, eye, testis and plasma. Considering that the bulk of these data emanate from animal studies, it is worthwhile to perform clinical studies that investigate if this antioxidant effect of honey can be extrapolated to human subjects with chronic diseases. The molecular structures of some of the biologically active constituents such as fructose and oligosaccharides which may play a role in the hypoglycemic or antidiabetic effect of honey are shown in Figure 1 [21,22,23,24,25]. 

## 2. Honey: A Novel Antioxidant–Evidence from *in Vitro* Studies

**Figure 2:**
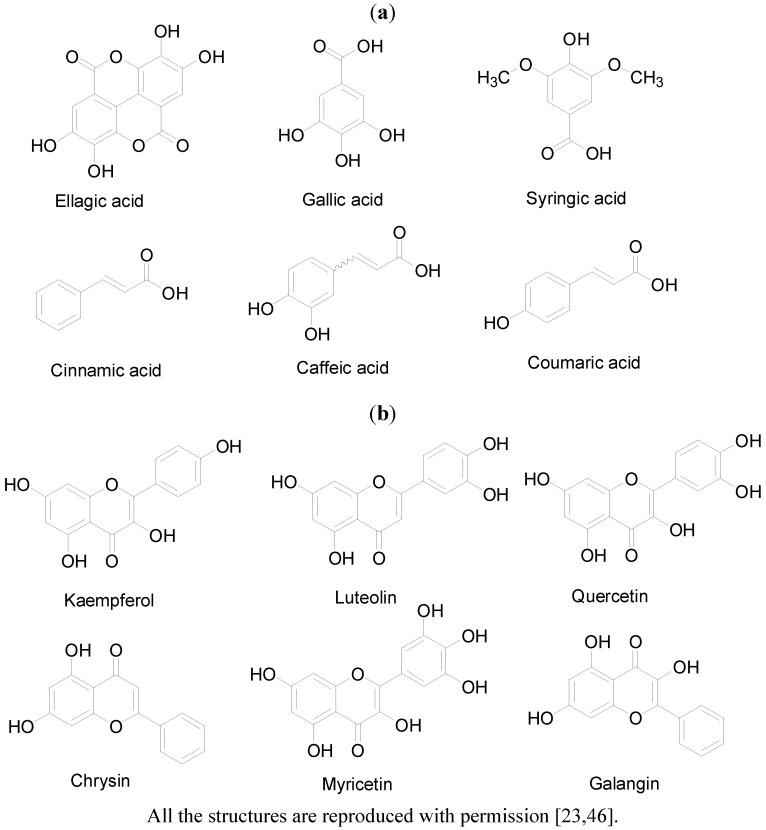
Molecular structures of some of the organic acids, flavonoids and phenolic compounds present in honey.

The *in vitro* antioxidant properties of natural or synthetic agents are measured in the form of antiradical activity using the 1,1-diphenyl-2-picrylhydrazyl (DPPH) scavenging assay, oxygen radical absorbance capacity (ORAC) assay and ferric reducing antioxidant power (FRAP) assay [26,27]. Using these tests, different varieties of honey from various countries and geographical regions have been shown to exhibit high antioxidant properties. Turkish red pine honey produced by *Marchalina hellenica *was reported to scavenge DPPH effectively, suggestive of its antiradical activities [28]. Some Saudi Arabian honey samples were demonstrated to exhibit antioxidant activities [29]. Similar antioxidant properties were also reported for Peruvian honey [30]. Australian honey produced by the stingless bees *Trigona carbonaria* was reported to have high antioxidant properties [31]. Malaysian tualang honey produced by the giant Asian bees *Apis dorsata *has been shown to exhibit good antioxidant and antiradical activities [32,33,34]. Antioxidant activities have also been documented for American buckwheat honey [35], Croatian oak honeydew honey [36], Spanish honey [37], Portugal honey [38], Cuban honey [39], Venezuelan honey [40] and Ecuadorian honey [41]. The antioxidant activity of honey is generally attributed to its phenolic compounds and flavonoids [33,35,42,43]. Findings from a recent study suggest that gamma irradiation may increase the antioxidant capacities and total phenolic contents in honey [27]. The main phenolic and flavonoid compounds in honey include ellagic acid, gallic acid, syringic acid, benzoic acid, cinnamic acid, ferulic acids, myricetin, chlorogenic acid, caffeic acid, hesperetin, coumaric acid, isoramnetin, chrysin, quercetin, galangin, luteolin and kaempferol [19,27,44,45]. While some of these bioactive compounds such as alangin, kaempferol, quercetin, isorhamnetin and luteolin are found in most honey samples, others such as hesperetin and naringenin are found in few honey varieties [45]. Among some Malaysian honey samples investigated, catechin was found to be a common flavonoid [33]. By and large, the avalanche of data on the *in vitro* antioxidant activities of honey indicate that honey is not only an antioxidant but also a rich source of antioxidants. The molecular structures of some of the biologically active ingredients or constituents including organic acids, flavonoids and phenolic compounds in honey which may contribute to the antioxidant effect of honey are presented in Figure 2a and b [23,46].

## 3. Honey: A Novel Antioxidant–Evidence from *in Vivo* Studies

Available data indicate that honey, like other antioxidant agents, does protect against damage or injury. This protective effect of honey is partly mediated via amelioration of oxidative stress in tissues such as GIT, liver, kidney, pancreas, eye, plasma, red blood cells and reproductive organs [11,12,13,14,15,19]. Therefore, the *in vivo* antioxidant effect of honey will be discussed in regard to its ability to ameliorate oxidative stress in various cells, tissues, organs or body fluids.

### 3.1. GIT

Evidence indicates that in diseased conditions such as diabetes mellitus, the GIT is susceptible to oxidative stress, which may impair the brush border membrane (BBM) fluidity [47]. The intestinal uptake of substances, molecules or mineral ions is also known to be modulated or influenced by the redox state of the transporter [48]. These transporters can be regulated rapidly through alteration of their trafficking or affinities in response to oxidative stress [49]. The beneficial effect of GSH, an antioxidant, on these transporters has been demonstrated [49]. It is suggested that such modification of redox or oxidative state might influence the bioavailability of essential macronutrients and drugs [49]. Antioxidant vitamin E was reported to increase the activities of CAT and SOD and reduce lipid peroxidation and protein oxidation in small intestine of diabetic rats [50]. At the moment, there is no evidence to suggest that honey exerts an antioxidant effect in the intestine. However, considering the reported antioxidant effect of honey in different cells or tissues such as pancreas, serum, kidney and liver [15,20,42,44], it is possible that honey might also ameliorate intestinal oxidative stress. Hence, it may not be a surprise that co-administration of hypoglycemic agents and honey further improves glycemic control in diabetic rats [51]. This might be due to modification of intestinal redox or oxidative state which influences the bioavailability of hypoglycemic drugs [49].

Available evidence indicates that honey exerts gastroprotective effect in rodents administered indomethacin, ethanol, aspirin or ammonia [11,52,53,54]. Although the sugars such as fructose, glucose, sucrose and maltose present in honey may play a role in its gastroprotective effect [11,53], there is a possibility that the antioxidant effect of honey may also contribute to its gastroprotective effect. This assertion is based on the evidence which indicates that increased mucosal lipid peroxide and reduced GSH levels exacerbate gastric hemorrhagic ulcer in diabetic rats [55]. Another study also reported increased lipid peroxidation and impaired antioxidant enzymes (increased SOD and decreased CAT activities) in gastric mucosa of rats with cold restraint stress-induced gastric ulceration [56]. A similar increase in oxidative stress level was also demonstrated in patients with peptic ulceration and gastric carcinoma [56]. Additional evidence in support of the role of antioxidant effect of honey in mediating its gastroprotective effect is provided by Kim [57]. The author found that oxidative stress is one of the mechanisms by which *Helicobacter pylori* induces gastric injury [57]. Interestingly, honey also inhibits the growth of *Helicobacter pylori*, which causes gastric and duodenal ulcers [58,59]. Considering the prolonged gastric ulcer healing, the prevalence of silent gastric ulcer or erosions and the highly oxidative milieu in diabetes mellitus [60,61,62], the antioxidant-mediated gastroprotective effect of honey might be beneficial in diabetes mellitus. Honey might also be valuable in ulcerative colitis [63]. In trinitrobenzosulphonic acid (TNBS)-induced ulcerative colitis model in rats, honey combined with an antibiotic (sulfasalazine) enhanced antioxidant defense system and reduced oxidative damage and colonic inflammation [63]. Similarly, in rats with chemically (TNBS)-induced colitis, honey supplementation significantly reduced the colonic mucosal malondialdehyde (MDA) content [64]. In a nutshell, amelioration of oxidative stress as a result of honey administration might restore impaired intestinal brush border membrane (BBM) fluidity and redox or oxidative state. This might modify or modulate the bioavailability of vital macronutrients and drugs. This antioxidant effect in the GIT might also contribute to the gastroprotective effect of honey.

### 3.2. Livers

The liver plays an important role in many metabolic processes such as glycemic control, detoxification of xenobiotics, synthesis of lipoproteins, hormones and enzymes [65]. In diabetes mellitus, the liver is associated with abnormalities such as elevations in serum aspartate aminotransferase, alkaline phosphatase and alanine aminotransferase [66]. Available evidence suggests that the liver is susceptible to oxidative stress and damage; and the beneficial effect of antioxidants on hepatic oxidative stress has been documented [67,68]. In the liver of young and middle-aged rats, honey supplementation was reported to restore activities of CAT and GPx [69]. In male BALB/c mice administered trichlorfon, oral supplementation with pine honey restored the activities of hepatic GPx (significantly) and SOD and CAT (moderately) and reduced hepatic damage [45]. Similar hepatoprotective effect of honey was also reported in STZ-induced diabetic rats [70] and sheep administered carbon tetrachloride (CCl_4_) [71]. In rats with obstructive jaundice, usually associated with increased hepatic ROS formation, oxidative stress and inflammation [72,73], honey supplementation significantly reduced the levels of MDA and increased GSH content in the liver [74]. The amelioration of oxidative stress, as a result of honey administration, was accompanied by significant reductions in the size of enlarged hepatocytes and edema, restoration of bile canaliculi dilatation and reduced number of apoptotic cells [74]. Similar hepatoprotective effect of honey was also reported in rats with obstruction of the common bile duct [75]. In rats with N-ethylmaleimide (NEM)-induced liver injury, honey supplementation significantly restored the levels of hepatic glutathione, ameliorated the (NEM)-induced congestion and mononuclear cell infiltration in the liver [76]. Supplementation with honey and ginseng was reported to protect against CCl_4_-induced hepatotoxicity in rats by reducing lipid peroxidation and enhancing antioxidant capacity [77]. These findings, generally, suggest that amelioration of oxidative stress in the liver may contribute to the hepatoprotective effect of honey. 

### 3.3. Pancreas

The pancreas plays an important role in glucose homeostasis [65]. Evidence indicates that the efficiency of pancreas to secrete insulin declines in diabetes mellitus, resulting in deterioration of glycemic control [78]. The role of oxidative stress is implicated in the decline of pancreatic function in diabetes mellitus [78,79]. The pancreatic β-cells are highly vulnerable to oxidative stress as a result of their intrinsically low expressions and activities of free radical scavenging enzymes [80]. The beneficial effect of antioxidants in protecting the pancreas against oxidative stress and damage is well documented [81]. We have shown that honey has a potential to protect pancreas against oxidative stress and damage. Honey supplementation significantly reduced elevated levels of MDA and restored the activities of SOD and CAT in pancreas of diabetic rats [82]. We have also investigated and compared the effect of glibenclamide alone with that of glibenclamide and honey on oxidative stress in pancreas of diabetic rats [83]. The data revealed that glibenclamide did not ameliorate oxidative stress in the pancreas of diabetic rats. In contrast, the pancreas of diabetic rats treated with the combination of glibenclamide and honey had increased CAT activity and restored the elevated GPx activity and levels of MDA [83]. In another related study, we found that the combination of glibenclamide and metformin ameliorated oxidative stress only partially [84]. This was evidenced by the attenuation of GPx activity only while no significant effect on other antioxidant enzymes and lipid peroxidation was observed in the pancreas of diabetic rats [84]. In contrast, the combination of glibenclamide, metformin and honey significantly increased CAT activity while GPx activity was down-regulated. The combination with honey also considerably prevented lipid peroxidative damage [84]. These data clearly suggest that the combination of hypoglycemic agents with honey markedly restores antioxidant enzymes, ameliorates oxidative stress and protects the pancreas against oxidative damage.

### 3.4. Kidney

The kidney plays an important role in the excretion and regulation of osmolytes, especially in diabetes mellitus [85]. In hypertension, the kidney is an important regulator of many mechanisms involved in the regulation of blood pressure [85]. Thus, any damage to this organ will exacerbate these two diseases. Available evidence indicates that the kidney is a major target of oxidative stress in both diabetes mellitus and hypertension [85,86].

#### 3.4.1. Diabetes Mellitus

Without adequate treatment, diabetic patients are more likely to develop diabetic nephropathy and overt renal failure [85]. In view of the evidence that implicates a role of oxidative stress in the complications of diabetes mellitus [86], a number of studies have investigated the potential role of antioxidants in protecting the kidney against oxidative damage [87]. Our studies have shown that honey ameliorates renal oxidative stress. Honey administration to diabetic rats significantly increased total antioxidant status (TAS), activities of glutathione S-transferase (GST), glutathione reductase (GR), CAT and GPx [15]. It also restored SOD activity while it reduced the levels of lipid peroxidation. This antioxidant effect of honey was accompanied by improvements in renal morphology as evidenced by reduced mesangial matrix expansion and thickening of glomerular basement membrane in the honey-treated diabetic rats [15]. The study further revealed that the antioxidant effect of the lowest dose of honey, 0.2 g/kg body weight (despite its lower hypoglycemic effect), was comparable to that of the higher doses (1.2 or 2.4 g/kg body weight) [15]. This suggests that the antioxidant effect of honey is not dependent on its hypoglycemic effect. Other studies have also demonstrated the antioxidant effect of honey in kidney of rats with diabetes [88] or without diabetes [89]. 

We have also investigated and compared the effects of honey, glibenclamide, metformin, glibenclamide and metformin, as well as their combinations with honey on oxidative stress in kidney of diabetic rats [90,91]. Our results indicated that the activities of CAT and GR as well as TAS and reduced glutathione (GSH) in diabetic rats treated with metformin and/or glibenclamide remained similar to those of diabetic control rats [91]. In contrast, metformin or glibenclamide combined with honey significantly increased the activities of CAT and GR and also TAS and GSH in the kidney of diabetic rats [91]. Other authors have also shown that in rats administered CCl_4_, honey supplementation prevented CCl_4_-induced nephrotoxicity via enhanced antioxidant capacity and reduced lipid peroxidation [77]. In summary, these results suggest that the combination of hypoglycemic drugs, glibenclamide and/or metformin with honey in diabetic rats ameliorates renal oxidative stress better than either agent administered alone [90,91]. 

#### 3.4.2. Hypertension

The role of oxidative stress in hypertension is a subject of much research interest. Oxidative stress is implicated in the pathogenesis of hypertension [92], while some evidence also indicates that hypertension generates oxidative stress [93]. These lines of evidence support a role of oxidative stress as an important determinant in the imbalance between vasoconstrictor and vasodilator mechanisms [92,93,94]. The beneficial effects of antioxidants in ameliorating oxidative stress and suppressing or reducing elevated blood pressure in experimental and clinical studies further corroborate the role of oxidative stress in hypertension [95]. In a recent study, we reported that honey supplementation in spontaneously hypertensive rats (SHR) restored the elevated antioxidant defenses (GST, TAS and CAT) in kidney of SHR [94]. Honey administration also prevented the formation of MDA in the kidney of SHR [94]. The amelioration of oxidative stress was accompanied by suppressed elevations in blood pressure in SHR [94].

The combination of diabetes mellitus and hypertension is associated with increased cardiovascular risk factors [96,97]. Besides other factors, evidence suggests that diabetes mellitus may exacerbate hypertension via increased oxidative stress [98]. We recently investigated the effect of honey on oxidative stress in kidney of rats with both diabetes mellitus and hypertension [99,100]. We found that honey supplementation significantly increased intracellular GSH, GSH/GSSG (oxidized glutathione) ratio, TAS and activities of GPx and GR in kidney of diabetic SHR [99,100]. The study also revealed that the antioxidant effect of honey resulted in further reductions in blood pressure of diabetic SHR but not of diabetic WKY [99,100]. These findings reveal that similar to the antioxidant effect of honey in the diabetic kidney, honey supplementation also ameliorates oxidative stress in the kidney of hypertensive rats. This antioxidant effect of honey results in suppression of blood pressure. Honey also exerts its antioxidant effect in kidney of rats with both diabetes mellitus and hypertension. We and others reported recently that reduced or impaired Nrf2 activity or expression contributes to increased susceptibility of kidney to oxidative stress in rats with chronic renal failure [101] or hypertension [94,102]. Data from our laboratory indicate honey may ameliorate oxidative stress via up-regulation of Nrf2 activity or expression [94,102]. Nrf2 is a transcription factor released from its repressor (Keap1) under oxidative or xenobiotic stress [103]. It translocates from the cytoplasm to the nucleus and binds to the antioxidant response element (ARE) in the promoter region of cytoprotective genes, resulting in their transcription [103]. The transcription of these genes subsequently induces free radical scavenging enzymes and other detoxifying enzymes which swiftly neutralize, detoxify and eliminate the oxidants or xenobiotics [103].

### 3.5. Plasma/Serum

In diabetes mellitus, the levels of plasma glucose, fructosamine and glycosylated hemoglobin are used as indicators of glycemic control [104]. Elevated plasma glucose generates ROS which cause oxidative stress [86]. Oxidative stress is implicated in the cellular dysfunction and complications of diabetes [86]. Evidence indicates that agents (such as pyridoxine, pyridoxamine and gliclazide) that scavenge or inhibit ROS formation enhance antioxidant defenses (SOD, thiols, TAS), reduce oxidative stress markers (8-isoprostanes and MDA) and protein glycosylation in diabetes [105,106]. In some studies, amelioration of oxidative stress using antioxidants was accompanied by reduced hyperglycemia [81]. However, some studies found no such effect [67]. Antioxidants have been shown to reduce the elevated levels of plasma glucose and glycosylated hemoglobin [81]. In STZ-induced diabetic Sprague-Dawley rats, honey supplementation reduced hyperglycemia [15,88]. A similar hypoglycemic effect of honey was also reported in alloxan-induced diabetic rats [107], STZ-induced diabetic Wistar-Kyoto rats [99] and STZ-induced diabetic SHR [99]. In those studies, similar to what was reported for other antioxidants, hypoglycemic effect of honey was accompanied by amelioration of oxidative stress [15,88,99].

Besides the hypoglycemic effect of honey, we also found in another study that honey supplementation reduced fructosamine concentrations in STZ-induced diabetic rats [51]. Fructosamine is a stable compound formed from the reaction of a carbonyl group of glucose with an amino group of protein [108]. It can be formed from serum proteins such as albumin to produce glycated serum protein. In a highly oxidative environment (such as in diabetes), fructosamine can form advanced glycation end products (AGEs), which is implicated in diabetic complications [108]. The ability of honey to reduce fructosamine may be attributed to its antioxidant effect. Honey has been shown to increase serum antioxidant capacity [103]. Compelling evidence indicates that glycation and oxidative reactions (or their products) are mutually dependent and strongly correlate [108,110]. A number of antioxidants such as α-lipoic acid, taurine, vitamins C and E have been shown to reduce fructosamine and glycosylated hemoglobin by inhibiting the formation of MDA, protein glycation and advanced glycation end products [108,111,112]. Another study reported reduced glycosylated hemoglobin in non-diabetic rats after chronic (52 weeks) honey supplementation [113]. A similar beneficial effect of honey was reported in patients with impaired lipid metabolism [114,115,116]. In diabetes, the antioxidant effect of honey may also be beneficial in lipid metabolism [51,113,114,115,116] (e.g., in inhibiting or preventing the oxidation of low density lipoprotein) [105]. A study that investigated the protective effect of honey and Nigella grains against methylnitrosourea-induced oxidative stress and carcinogenesis indicated that *Nigella sativa* grains reduced the elevated levels of MDA and nitric oxide (NO) in serum and produced a protective effect of 80%. On the other hand, combination of honey and *Nigella sativa* abolished the increases in MDA and NO and exerted a protective effect of 100% against MNU-induced oxidative stress and carcinogenesis [117]. Other studies have also demonstrated the antioxidant effect of honey in serum as evidenced by the increased plasma NO metabolites in healthy sheep [118] and the increased GPx activity and NO in the serum of alloxan-induced diabetic rats [119]. 

### 3.6. Reproductive Organs

The exposure to cigarette smoke (CS) causes apoptosis and damage in the testis [120]. Evidence implicates the role of oxidative stress in CS-induced testicular damage [121]. A number of studies have demonstrated the beneficial effects of antioxidants in preventing or ameliorating testicular damage in rodents [122]. A study investigated the effect of honey in the testis of rats exposed to CS [123]. It was found that honey supplementation for 13 weeks markedly reduced the level of lipid peroxidation [123]. Honey administration also increased the reduced TAS and restored the activities of SOD, GPx and CAT. This antioxidant effect of honey was associated with amelioration of testicular damage as evidenced by higher Leydig cell count, reduced percentage of tubules with germ cell loss, larger seminiferous tubules diameter and epithelial height [123]. These data suggest that honey may protect or ameliorate CS-induced testicular damage in rats via its antioxidant effect. The authors in one of their previous studies also reported that honey supplementation in normal rats improved spermatogenesis [13]. A recent study also demonstrated the beneficial effects of honey on sperm motility and morphology in rats [124]. A study by Abdul-Ghani and colleagues also indicated that honey supplementation in rats caused increased epididymal sperm count and improved the activity of testicular marker enzymes for spermatogenesis, as evidenced by increased sorbitol dehydrogenase and reduced lactate dehydrogenase [125]. Available data in ovariectomised female rats also suggest that honey may produce beneficial effects in female reproductive organs [14]. A similar beneficial effect of honey on oxidative stress was also reported in human subjects [126]. A study investigated the effects of 8-week honey supplementation on seminal plasma cytokines, oxidative stress and antioxidants in male road cyclists during intensive cycling training [126]. The study found that honey supplementation significantly increased the concentrations of seminal SOD, CAT and TAS. This antioxidant effect of honey was also associated with lower elevations in the seminal IL-1beta, IL-6, IL-8, TNF-alpha, ROS and MDA levels [126]. 

### 3.7. Eye

Visual impairment caused by alkali burns of the corneal and conjunctival surface is considered one of the most devastating injuries to the eye [127,128]. These chemical burns-induced eye injuries are accompanied by increased oxidative stress [127,128]. The beneficial effects of antioxidants in the treatment of these eye injuries have been reported [127,128]. A study investigated and compared the antioxidant effects of honey and conventional treatment in alkali injury on the eyes of New Zealand White rabbits [129]. The study did not find any significant difference in TAS and MDA levels in aqueous humour, vitreous humour and serum of rabbits treated with honey and conventional treatment [129]. The lack of significant effect of honey on oxidative stress parameters might be a result of the short duration of the study (7 days). Other possible explanations include non-inclusion of control and small sample size. Therefore, considering the previous data on the beneficial effects of antioxidants in chemical burns-induced eye injuries in animals [127,128], together with the limitations of this recent study [129], it is premature to conclude that honey is not beneficial in the treatment of chemical burns-induced eye injuries in animals. This is in view of the fact that honey has been shown to be beneficial in other eye diseased conditions such as in human patients with dry eye syndrome [130] or endophthalmitis [131]. 

### 3.8. Other Antioxidant Effects of Honey

In the erythrocytes of young and middle-aged rats, honey supplementation was reported to restore activities of CAT and GPx [69]. Evidence also indicates that honey has a potential to ameliorate oxidative stress in the brain and heart [39]. In a cultured endothelial cell line, Beretta and colleagues used cumene hydroperoxide to generate free radicals and oxidative stress [42]. The authors found that honey produced strong scavenging activity against lipophilic cumoxyl and cumoperoxyl radicals, reduced intracellular ROS generation and restored intracellular GSH [42]. The authors also reported that honey considerably inhibited oxidation of cell membrane and prevented cellular damage. The study further revealed that the antioxidant effect of honey was due to its phenolic acids and flavonoids [42]. The antioxidant effect of honey might also contribute to other beneficial effects of honey such as reduced weight gain and improved lipid metabolism in rats or human subjects administered honey [132,133,134].

### 3.9. Effects of Honey on Inflammation

Oxidative stress and inflammation are frequent manifestations and play an important role in the etiology of many diseases and disorders [74,76,135]. Evidence indicates that they are intimately interrelated as each can cause the other [74,76,135]. In rats with inflammatory bowel disease, intra-rectal honey administration significantly reduced myeloperoxidase (MPO) activity [64]. This was associated with lower levels of colonic MDA with no change in NO content [64]. A recent study that investigated the effects of honey and its extracts in rat models of inflammation reported that honey and its extracts inhibited NO and prostaglandin E(2) production [136]. The authors also found that honey and its extracts reduced edema and pain in inflammatory tissues. The inhibition of edema and pain was found to correlate with the inhibition of nitric oxide and prostaglandin E(2) [136]. Another study investigated the effects of various doses of honey on acute and chronic inflammations in rats using carrageenan, cotton pellet and formaldehyde methods and NO production by administering N^G^-nitro-L-arginine methyl ester (L-NAME) and L-arginine [137]. Honey supplementation was found to reduce the paw size, the granuloma weight and the arthritis in the carrageenan, the cotton pellet and formaldehyde methods, respectively [137]. Additional evidence in support of the anti-inflammatory effect of honey was demonstrated by inhibition of paw oedema by L-NAME and the loss of anti-inflammatory effect of honey following the administration of L-arginine [137]. These data indicate that honey can exert an anti-inflammatory effect via inhibition of NO and prostaglandin E(2) production and release [137]. This antioxidant effect may contribute to its anti-inflammatory effect. Table 1 summarizes the antioxidant effect of honey in different tissues.

**Table 1:** Summary of the antioxidant effects of honey in different tissues.

Tissue/Study design	Oxidative stress status		Ref.
	Control	Honey	
**GIT**			
Rats with TNBS-induced colitis	↑ MDA; ↑ MPO; ↓ SOD; ↓ CAT; ↓ GPx and ↓ GSH	↓ MDA; ↓ MPO; ↑ SOD; ↑ CAT; ↑ GPx and ↑ GSH	63,64
**Liver**			
Rats or mice with trichlorfon-, NEM- or CCl_4_-induced liver injury or obstructive jaundice	↑ GPx; ↑ CAT; ↓ GSH; ↑ MDA and TAC	↓ GPx; ↓ CAT; ↑ GSH; ↓ MDA and TAC	44,69,74,76,77
**Pancreas**			
Rats with STZ-induced diabetes	↑ SOD; ↑ GPx; ↓ CAT and ↑ MDA	↓ SOD; ↑ CAT; ↓ GPx and ↓ MDA	82,83,84
**Kidney**			
Rats with STZ-induced diabetes (diabetic SD) or with CCl_4_-induced nephrotoxicity	↑ MDA; ↓ TAS; ↓ CAT; ↓ GPx; ↓ GST; ↓ GR; ↑ SOD and ↓ GSH	↓ MDA; ↑TAS; ↑ CAT; ↑ GPx; ↑ GST; ↑ GR; ↓ SOD and ↑ GSH	15,77,88,90,91
Rats with hypertension (SHR)	↑ MDA; ↑ GST; ↑ TAS and ↑ CAT	↓ MDA; ↓ GST; ↓ TAS and ↓ CAT	94,102
Rats with diabetes (diabetic WKY)	↔ MDA; ↔ CAT; ↑ GPx; ↔ GR; ↓ TAS and ↔ GSH/GSSG	↔ MDA; ↔ CAT; ↔ TAS; ↓ GPx; ↓ GR and ↑ GSH/GSSG	99,100
Rats with both diabetes and hypertension (diabetic SHR)	↔ MDA; ↓ CAT; ↓ GPx; ↓ GR; ↓ TAS; ↔ GSH and ↔ GSH/GSSG	↔ MDA; ↔ CAT; ↑ GPx; ↑ GR; ↑ TAS; ↑ GSH and ↑ GSH/GSSG	99,100
**Plasma/serum**			
MNU-induced oxidative stress	↑ MDA and ↑ NO	↓ MDA and ↑ NO	117
Alloxan- or STZ-induced diabetic rats or non-diabetic rats	↓ GPx; ↓ NO and ↑ formation of glycated products (fructosamine and glycated hemoglobin)	↑ GPx; ↑ NO; ↑ TAS and ↓glycated products (fructosamine and glycated hemoglobin)	44,51,69,119
**Reproductive organs**			
Testis of rats exposed to cigarette smoke	↑ MDA; ↓ TAS; ↓ SOD; ↓ CAT and ↑ GPx	↓ MDA; ↓ GPx; ↑ TAS; ↑ SOD; ↑ CAT and ↑ GSH	123
Seminal oxidative stress in male cyclists undergoing intensive cycling training	↓ TAS; ↓ SOD and ↓ CAT	↓ MDA; ↓ ROS; ↑ SOD, ↑ CAT and ↑ TAS	126
**Other tissues or cells**			
Whole blood and erythrocytes of young (2 months) and middle-aged (9 months) rats	Whole blood: ↑ DNA damage; Erythrocytes: ↓ GPx and ↑ CAT	↓ DNA damage ↑ GPx and ↓ CAT	44,69
In a cultured endothelial cell line	↑ ROS and ↓ GSH	↓ ROS and ↑ GSH	42
In inflammation	↑ NO and ↑ prostaglandin E(2)	↓ NO; ↓ prostaglandin E(2) and ↓ inflammation	136,137

TNBS, trinitrobenzene sulfonic acid; MPO, myeloperoxidase; NEM, N-ethylmaleimide; TAC or TAS, total antioxidant capacity or status; 8-IP, 8-isoprostane; SOD, superoxide dismutase; CAT, catalase; GPx, glutathione peroxidase; GR, glutathione reductase; glutathione S-transferase; NO, nitric oxide; ROS, reactive oxygen species; GSH, reduced glutathione; GSSG, oxidized glutathione; MDA, malondialdehyde; ↑ = increased/enhanced; ↓ = reduced/attenuated; ↔ = no significant effect.

## 4. Could Honey Be a Better Antioxidant than Some of the Commonly Available Antioxidants

The beneficial effects of different antioxidants, especially vitamins C and E, are well documented in various disease models in both rodents and humans [138,139,140,141,142]. However, some demerits have also been reported for some of these antioxidants or vitamins [143]. Although they are potent, their mechanisms of antioxidant action are a bit complex in the sense that their antioxidant action does not end with scavenging or elimination of free radicals [143]. Instead, these vitamins themselves become pro-oxidants that may require antioxidants for their regeneration into the active or antioxidant form [144,145]. This, therefore, suggests that supplementation with such antioxidants especially at high doses may perturb the delicate physiological balance among the antioxidants [142,144,145]. In diseases characterized by oxidative stress, such an imbalance in endogenous antioxidant defenses caused by exogenously administered or ingested antioxidants, instead of ameliorate may further exacerbate oxidative stress or may do more harm than good [142,146]. In some cases, these vitamins exacerbated diseases and increased mortality [147]. Most of those trials used vitamins C and E as antioxidants of first choice and were characterized by undefined selection of doses. For instance, there are reports that large doses of dietary α-tocopherol supplementation interfere with or displace γ-tocopherol in the plasma and tissues [143,148] or may enhance formation of tumor [144,149]. Antioxidant (β-carotene) supplementation in smokers was also reported to exacerbate the risk of cancer [147]. Although both α-tocopherol and γ-tocopherol protect against peroxynitrite-induced lipid oxidation, evidence indicates that γ-tocopherol is essential for efficient removal of peroxynitrite-derived nitrating species [145,150]. Some evidence also suggests that γ-tocopherol is a better inhibitor of nitrogen dioxide-mediated nitrosation than α-tocopherol [151]. 

In a nutshell, as a result of dearth of data, it remains unclear if honey will be more effective or efficacious than vitamins C or E in ameliorating oxidative stress. However, honey administered at therapeutic doses is likely to be devoid of pro-oxidant properties often associated with vitamins C and E. Another advantage of honey over vitamins C and E is the fact that honey comprises several bioactive constituents. Many of these constituents may produce synergistic antioxidant effects. Besides, unlike vitamin C or E which requires the other for regeneration into the active form [138,139], this may be unnecessary with the use of honey. If any of the antioxidant constituents in honey exhibits pro-oxidant properties, there would be sufficient antioxidants for its regeneration. Available evidence suggests that honey may ameliorate oxidative stress by scavenging both free radicals such as OONO^−^, O_2_^•−^[38] and non-free radicals such as NO [64]. Recently, we found that honey ameliorates oxidative stress by up-regulating Nrf2 moderately, an important intracellular transcription factor [88]. Evidence also suggests that honey may reduce inflammation as evidenced by the inhibition of NO and prostaglandin E(2) production [64,136]. This is important because both oxidative stress and inflammation are interrelated [135]. Besides, evidence implicates the role of oxidative stress and inflammation in the pathogenesis and complications of many chronic diseases such as diabetes mellitus and hypertension [152]. Therefore, considering the antioxidant and anti-inflammatory effects of honey [15,74,82,90,91,94,99,100,136,137], the use of honey might be more beneficial or advantageous than some of the previously investigated antioxidants such as vitamins C and E.

## 5. Conclusions

The prevalence of chronic diseases such as diabetes mellitus, hypertension, atherosclerosis and cancer in the population is increasing. This has also resulted in increased mortality resulting from these diseases. The evidence which implicates the role of oxidative stress in the pathogenesis or complications of these diseases suggests that antioxidants may be beneficial. The data from large scale clinical trials have not been impressive and a number of reasons have been posited. One is the choice of certain antioxidants over the others – primarily, the use of vitamins C and E only in most of the trials. Honey is a natural product with many health benefits. The data presented in this paper indicate that honey may or can ameliorate oxidative stress in the GIT, liver, pancreas, kidney, testis and plasma. Besides, the data also indicate that the combination of conventional therapy, e.g., antidiabetic drugs, with honey produces synergistic antioxidant effect in the pancreas, kidney and plasma of diabetic rats. Honey, administered alone or in combination with conventional therapy, might be a novel antioxidant in the management of many diseases associated with oxidative stress. There is no doubt that studies investigating the effect of honey on oxidative stress are at a relatively early stage. However, a closer look at the presented data reveals that clinical studies that investigated the antioxidant effect of honey are limited. Therefore, it will be worthwhile to investigate some of these data in clinical studies to confirm if this antioxidant effect of honey can be extrapolated to humans. Together with its anti-inflammatory effect, the use of honey (especially in combination with conventional therapy) in the management of chronic diseases associated with oxidative stress holds much promise.
